# Neural stem and progenitor cell fate transition requires regulation of Musashi1 function

**DOI:** 10.1186/s12861-015-0064-y

**Published:** 2015-03-18

**Authors:** Angus M MacNicol, Linda L Hardy, Horace J Spencer, Melanie C MacNicol

**Affiliations:** Department of Neurobiology and Developmental Sciences, University of Arkansas for Medical Sciences, 4301 W. Markham, Slot 814, Little Rock, AR 72205 USA; Winthrop P. Rockefeller Cancer Institute, University of Arkansas for Medical Sciences, 4301 W. Markham, Little Rock, AR 72205 USA; Department of Biostatistics, University of Arkansas for Medical Sciences, 4301 W. Markham, Little Rock, AR 72205 USA; Center for Translational Neuroscience, University of Arkansas for Medical Sciences, 4301 W. Markham, Little Rock, AR 72205 USA

**Keywords:** Musashi, Stem cell, Differentiation, Survival, mRNA translation, Cell cycle

## Abstract

**Background:**

There is increasing evidence of a pivotal role for regulated mRNA translation in control of developmental cell fate transitions. Physiological and pathological stem and progenitor cell self-renewal is maintained by the mRNA-binding protein, Musashi1 through repression of translation of key mRNAs encoding cell cycle inhibitory proteins. The mechanism by which Musashi1 function is modified to allow translation of these target mRNAs under conditions that require inhibition of cell cycle progression, is unknown.

**Results:**

In this study, we demonstrate that differentiation of primary embryonic rat neural stem/progenitor cells (NSPCs) or human neuroblastoma SH-SY5Y cells results in the rapid phosphorylation of Musashi1 on the evolutionarily conserved site serine 337 (S337). Phosphorylation of this site has been shown to be required for cell cycle control during the maturation of *Xenopus* oocytes. S337 phosphorylation in mammalian NSPCs and human SH-SY5Y cells correlates with the de-repression and translation of a Musashi reporter mRNA and with accumulation of protein from the endogenous Musashi target mRNA, p21^WAF1/CIP1^. Inhibition of Musashi regulatory phosphorylation, through expression of a phospho-inhibitory mutant Musashi1 S337A or over-expression of the wild-type Musashi, blocked differentiation of both NSPCs and SH-SY5Y cells. Musashi1 was similarly phosphorylated in NSPCs and SH-SY5Y cells under conditions of nutrient deprivation-induced cell cycle arrest. Expression of the Musashi1 S337A mutant protein attenuated nutrient deprivation-induced NSPC and SH-SY5Y cell death.

**Conclusions:**

Our data suggest that in response to environmental cues that oppose cell cycle progression, regulation of Musashi function is required to promote target mRNA translation and cell fate transition. Forced modulation of Musashi1 function may present a novel therapeutic strategy to oppose pathological stem cell self-renewal.

## Background

The extensive efforts directed towards identification of the gene transcription mechanisms controlling stem and progenitor cell differentiation have yielded a wealth of relevant information ([1-5] and references therein). Much less is known about the complimentary mRNA translation mechanisms that contribute to control of stem cell differentiation. The mRNA-binding protein, Musashi has been demonstrated to play a critical role in promoting stem cell self-renewal [6-8]. Musashi has been implicated in the progression of numerous cancers including glioma, medulloblastoma and neuroblastoma, where it has been proposed to promote the self-renewal of cancer cells with stem cell–like properties [9-19]. Musashi promotes both physiological and pathological stem cell self-renewal through the repression of translation of target mRNAs encoding proteins that mediate cell cycle arrest and differentiation e.g. p21^WAF1/CIP1^, Numb and others [10,20-24]. Musashi target mRNAs are de-repressed and translated in response to environmental signals that require cell cycle arrest, indicating that a mechanism exists to reverse the inhibitory action of Musashi [25-28]. However, the mechanism by which Musashi-mediated translational control function is regulated to effect a context-specific response in mammalian neural stem cells has not been determined [28].

Characterization of the mechanism controlling cell fate transition in maturing oocytes of the frog, *Xenopus laevis,* has suggested a candidate Musashi regulatory strategy. In this model, progesterone-stimulated oocyte maturation, like differentiation of neural stem and progenitor cells, requires the translation of Musashi target mRNAs [29,30]. Musashi1 is present in immature oocytes but does not direct translational activation until after progesterone stimulation. The functional switch to activate translation of Musashi1 target mRNAs in progesterone-stimulated oocytes requires regulatory phosphorylation that is mediated through cyclin-dependent kinase and extracellular-signal-regulated kinases (ERK/MAP kinase) signaling [31]. The sites of regulatory phosphorylation are conserved in the mammalian Musashi1 protein (corresponding to serine 312 and 337) [31]. Based on these observations, we hypothesized that regulatory phosphorylation of Musashi1 may be relevant to control of cell fate transitions in mammalian neural stem cells.

In this study, we report that regulatory serine 337 phosphorylation of mammalian Musashi1 occurs concomitant with de-repression and translation of target mRNAs. Inhibition of this regulatory Musashi phosphorylation attenuates the initiation of differentiation of NSPCs and of transformed SH-SY5Y neuroblastoma cells. We further demonstrate that Musashi1 is regulated in response to cell death signals and that inhibition of Musashi1 phosphorylation promotes aberrant survival of primary and transformed neural progenitor cells under conditions of nutrient deprivation. Together, our findings indicate a critical requirement for site-specific phosphorylation in the regulation of Musashi1 function during neuronal cell fate transitions.

## Results

**Figure 1:**
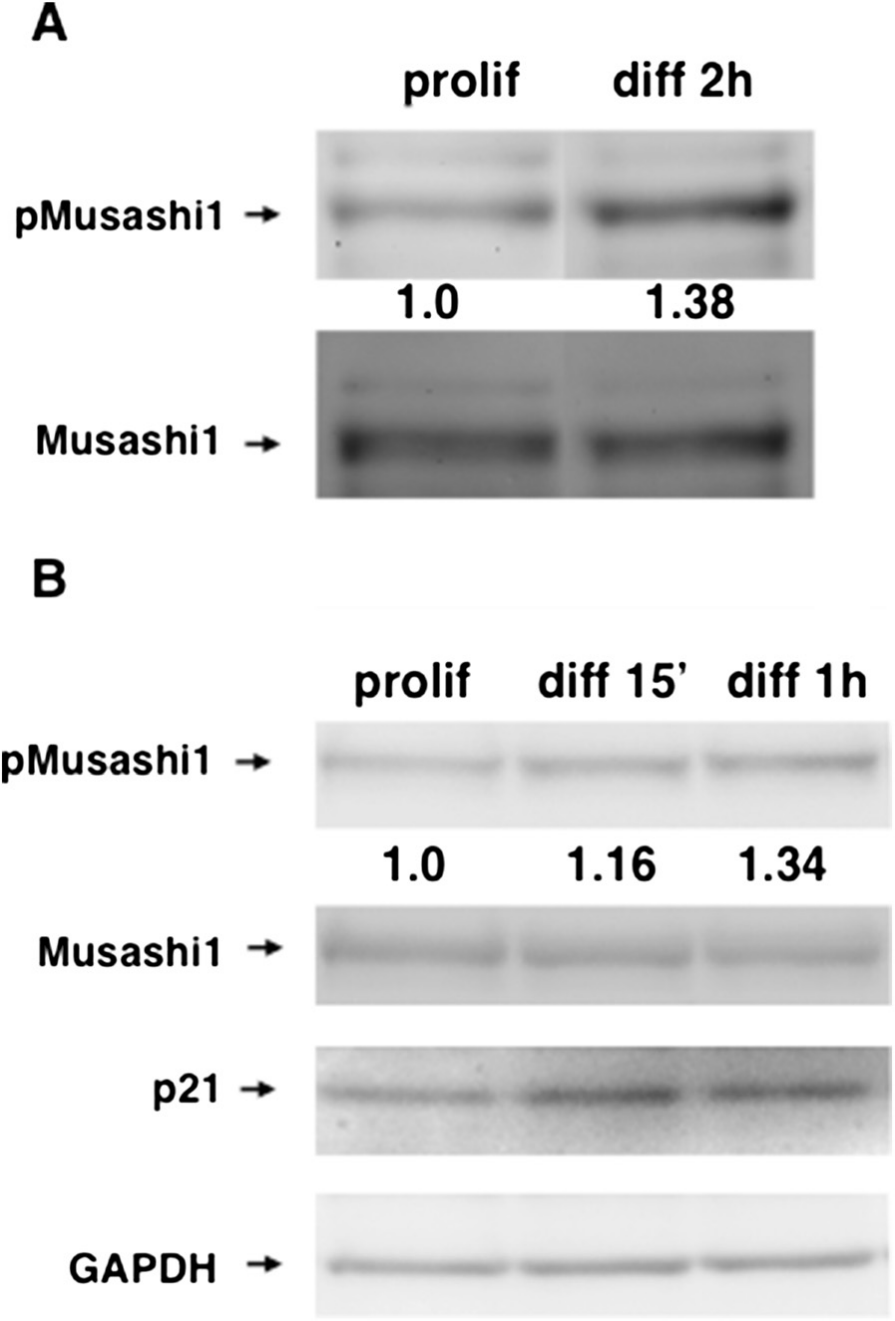
**Musashi undergoes regulatory phosphorylation in response to neural differentiation. (A)** Primary embryonic rat neural stem/progenitor cells (NSPCs) were maintained as neurospheres in proliferation media (prolif) or plated on adherent substrate in differentiation media for 2 hrs (diff). Protein lysates were processed for western blotting with the indicated antibodies. **(B)** Human neuroblastoma SH-SY5Y cells were maintained as neurospheres in proliferation media (prolif), or under differentiation conditions (diff) for the indicated times and analyzed by western blotting with the indicated antibodies.

We have recently demonstrated that regulatory phosphorylation of Musashi1 is required to allow the target mRNA translation that mediates cell cycle control during maturation of *Xenopus* oocytes [31]. To determine if Musashi1 is similarly regulated in neural stem and progenitor cells, we examined the phosphorylation state of the conserved serine 337 (S337, the equivalent of the *Xenopus* S322 site) in primary embryonic rat NSPCs using a phospho-specific antiserum that recognizes mammalian S337 [31]. A basal level of Musashi1 S337 phosphorylation was observed in control, proliferating neurosphere cultures that rapidly increased upon exposure to differentiation conditions (culture on adherent substrate in the presence of retinoic acid, Figure 1A). Over three independent experiments, the normalized ratios of phosphorylated Musashi1 were significantly increased (relative to levels of GAPDH in the same sample); 1.16, 1.38 and 1.32, respectively with a mean of 1.28 (95% confidence interval: 1.03 to 1.61; p = 0.041, one sample t-test). A similar increase in the level of Musashi1 S337 phosphorylation was observed in transformed human SH-SY5Y neuroblastoma cells upon exposure to differentiation conditions (Figure 1B). Over three independent experiments, the normalized ratios of phosphorylated Musashi1 were significantly increased (relative to levels of GAPDH in the same sample); 1.41, 1.34 and 1.58, respectively with a mean of 1.44 (95% confidence interval: 1.17 to 1.78; p = 0.017, one sample t-test).

**Figure 2:**
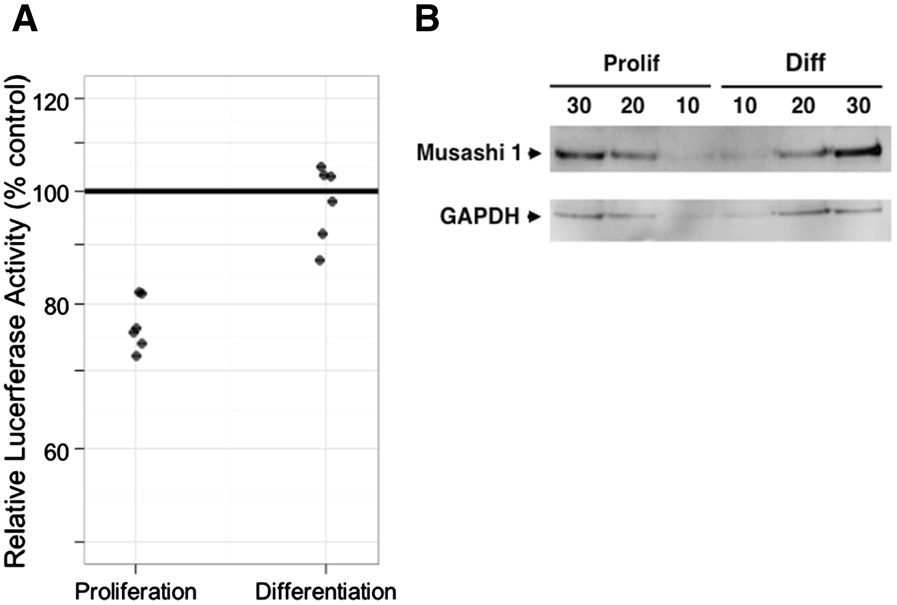
**De-repression of a Musashi-dependent reporter mRNA during differentiation of SH-SY5Y cells. (A)** SH-SY5Y cells were transfected with plasmids encoding luciferase reporter mRNAs under control of a Musashi binding element (MBE) or a control 3′ UTR (Control) and luciferase activity was assessed, relative to a co-transfected Renilla luciferase standard, following culture under proliferation or differentiation conditions for 24 hours. The dot plot shows the relative luciferase activity of six independent experiments as a percent of the 3′ UTR control (100%) for both proliferation and differentiation, as indicated. Luciferase expression was repressed in proliferating cells (mean of 77% with a 95% confidence interval: 73 to 81%; p < 0.0001, one sample t-test) but not in differentiated cells (mean of 97.7% with a 85% confidence interval 90.6 to 105.4%, p > 0.47, one sample t-test). **(B)** Western blot demonstrating persistence of Musashi1 in SH-SY5Y cells 24 hours after induction of differentiation. In this experiment, 10, 20 and 30 μg total protein (as indicted) were analyzed for Musashi1 and GAPDH protein levels from proliferating neurosphere culture (Prolif) or cells cultured in neuronal differentiation media for 24 hours (Diff). A representative experiment is shown.

Based on these findings, we hypothesized that the modest but significant increase in Musashi1 regulatory phosphorylation may facilitate the de-repression of the Musashi target mRNAs that are required to mediate cell cycle control during neural differentiation. The p21^WAF1/CIP1^ mRNA is a target of Musashi1 [20] that must be de-repressed to facilitate neural differentiation [25-27]. Musashi1 phosphorylation was coincident with an increase in p21^WAF1/CIP1^ protein levels, consistent with de-repression and translation of the p21^WAF1/CIP1^ mRNA (Figure 1B). As p21^WAF1/CIP1^ protein levels are influenced by multiple regulatory mechanisms [32], we utilized a Musashi-specific reporter mRNA to confirm that Musasahi1 regulatory phosphorylation correlated with target mRNA translation. The reporter mRNA encoded the Firefly luciferase gene under control of an mRNA 3′ untranslated region containing a Musashi-specific regulatory element (Musashi Binding Element, MBE) [28]. Translation of the reporter Firefly luciferase mRNA was repressed in proliferating SH-SY5Y cells (Figure 2A). Over six independent experiments, the levels of Firefly luciferase were significantly decreased (relative to levels expressed from a control reporter lacking an MBE); 82, 76, 76, 72, 74 and 82%, respectively with a mean of 77% (95% confidence interval: 73 to 81%; p < 0.0001, one sample t-test). Similar repression of this Musashi target reporter mRNA has been previously demonstrated in proliferating primary NSPCs [28]. Exposure of the SH-SH5Y cells to differentiation conditions for 24 hours resulted in the de-repression of the Musashi reporter mRNA back to levels indistinguishable from the control reporter lacking an MBE (Figure 2A); 104.5, 103, 98, 103, 91.6 and 87.6%, respectively with a mean of 97.7% (85% confidence interval 90.6 to 105.4%, p > 0.47, one sample t-test). The de-repression of the Musashi reporter mRNA is consistent with the increase in p21^WAF1/CIP1^ protein levels seen during SH-SY5Y differentiation (Figure 1B). The reporter mRNA was expressed at equivalent levels under each culture condition (data not shown) and importantly, the level of Musashi1 protein was not significantly decreased during early differentiation (24 hr, Figure 2B). While de-repression of target mRNAs could be mediated through degradation of the repressing mRNA-binding proteins, our findings indicate that the de-repression and translation of Musashi target mRNAs during initiation of differentiation can occur in the presence of Musashi protein. Musashi1 protein has been similarly shown to persist during the early phases of differentiation of P19 embryonic carcinoma cells [20], again suggesting that Musashi1 activity is regulated independently of protein degradation.

**Figure 3:**
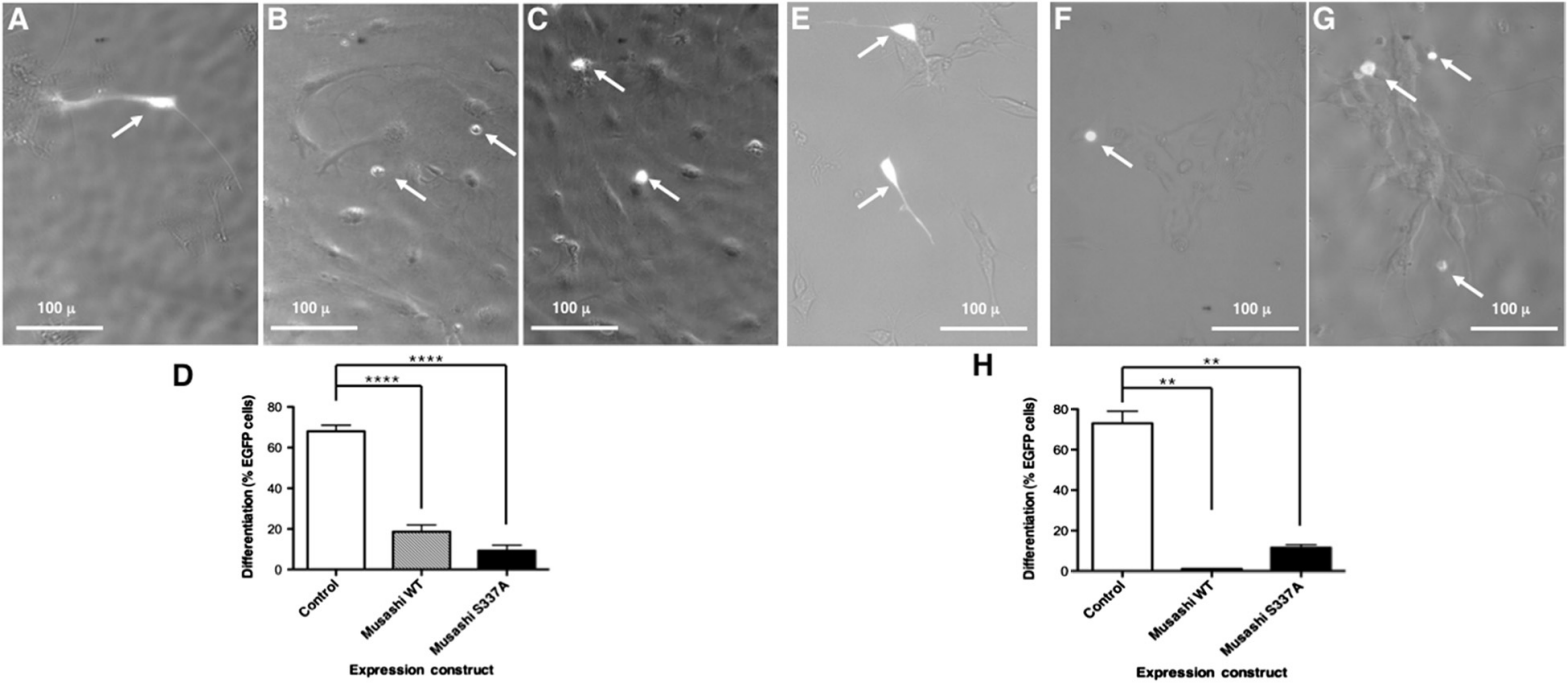
**Overexpression of Musashi1 blocks neural differentiation.** NSPCs **(panels A-D)** and SH-SY5Y cells **(panels E-H)** were transfected with a plasmid encoding the eGFP moity alone **(A and E)**, wild-type Musashi1 **(B and F)** or Musashi1 encoding a non-phosphorylatable S337A mutation **(C and F)** fused to a C-terminal eGFP epitope tag and incubated at low cell density under differentiation conditions for 4 days (NSPCs) or 7 days (SH-SY5Y) and scored for initiation of differentiation phenotype (hypertrophic cells with neurites > 2 soma lengths). Representative photos shown. Some variations in the intensity and subcellular distribution of Musashi1 or Musashi1 S337A were noted in transfected cells within the same experiment. Over the course of three experiments, a total of 70 NSPC cells **(A)** or 136 SHSY5Y cells **(E)** transfected with eGFP alone were counted. **(D and H)** represent histograms showing quantitation of the differentiation data (mean of three experiments with SEM) at 14 days **(D)** or 7 days **(H)**. ****,*p* < 0.0001 for **(D)**; **,*p* < 0.01 for **(H)**) as assessed by one-way ANOVA.

We next wished to determine if regulation of Musashi1 was required to mediate differentiation of neural stem and progenitor cells. To address this issue, we sought to inhibit Musashi1 regulation through expression of competitive, phospho-inhibitory mutant Musashi1 protein (where serine 337 was mutated to a non-phosphorylatable alanine, Musashi1 S337A). The mutant Musashi S337A was fused to eGFP for visualization and utilized to assess the role of regulatory phosphorylation in differentiation of NSPCs and SH-SY5Y cells. Control, eGFP-transfected NSPCs exhibited a high level of neurite extension after culture in differentiation conditions, (68% +/− 3 SEM cells with neurites > 2 cell body length; n = 3, Figure 3A and D). Initiation of differentiation was scored in transfected versus untransfected cells after 2 days of differentiation conditions, a time point when the cell density remained low enough to clearly distinguish morphological differentiation of isolated cells. Under these conditions, the level of control cells exhibiting differentiation was similar to the level of non-transfected cells exhibiting differentiation in the same culture dish. Expression of Musashi1 S337A resulted in a dramatic reduction in number of cells exhibiting phenotypic differentiation. Similarly, over-expression of the wild-type Musashi1 also inhibited differentiation, although to a somewhat lesser extent (Figure 3B and D, Musashi1; 18.7% differentiation +/− 3.3, Figure 3C and D, Musashi1 S337A; 9.3% differentiation +/− 2.7; SEM n = 3). An inhibitory effect of expression of Musashi1 S337A and wild-type Musashi1 was also observed upon initiation of differentiation by SH-SY5Y cells (Figure 3E and H, Control; 73% +/− 5% SEM neurite extension, Figure 3F and H, Musashi1; 1% +/− 1% SEM neurite extension, Figure 3G and H, Musashi1 S337A; 11.5% +/− 1.5% SEM neurite extension; n = 3). Taken together, our data indicate that attenuating the regulation of Musashi function interferes with initiation of neural differentiation.

**Figure 4:**
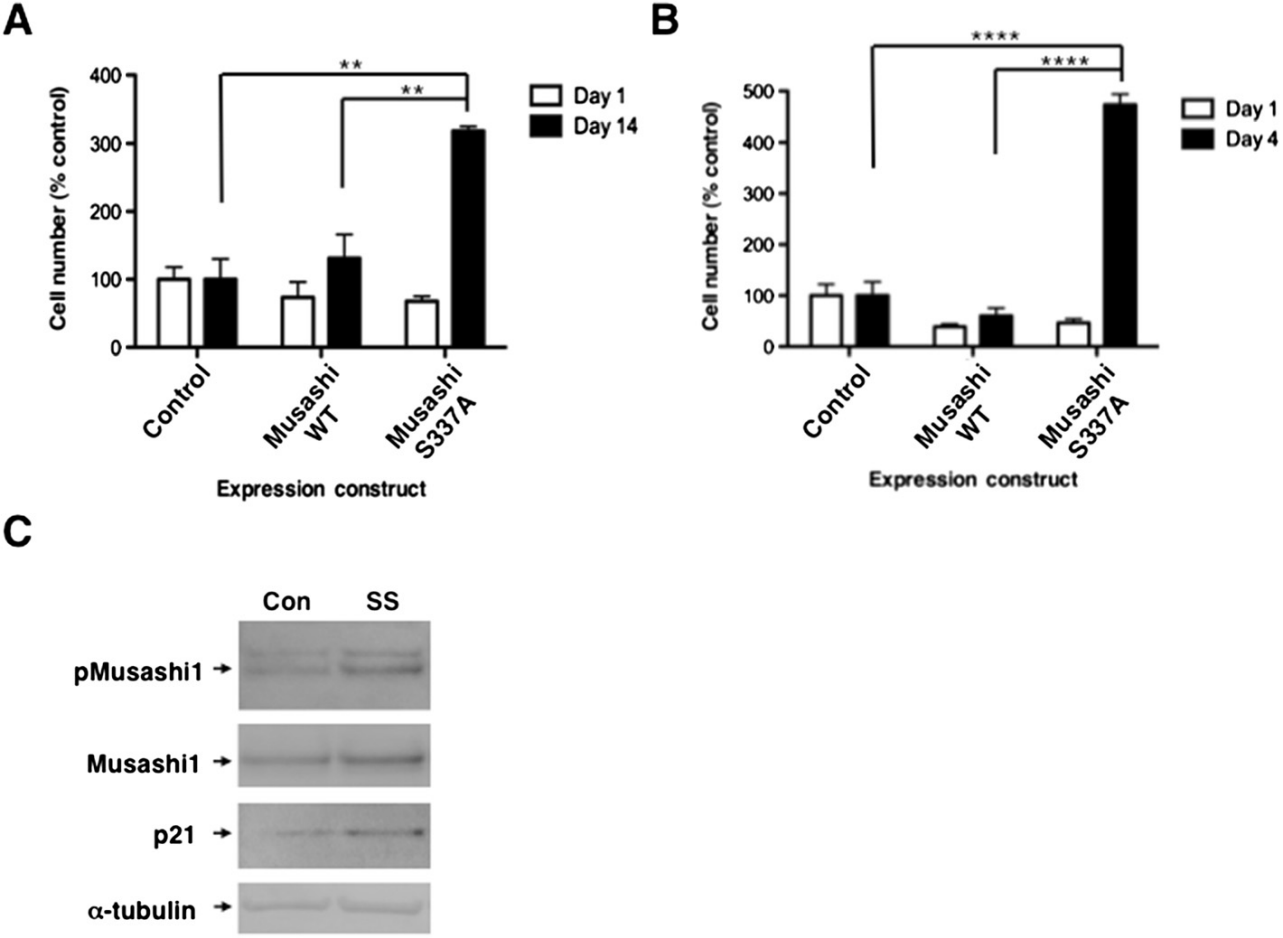
**Inhibition of Musashi phosphorylation promotes cell survival. (A)** NSPCs and **(B)** SH-SY5Y cells were transfected with the eGFP tagged Musashi proteins described in Figure 3 and fluorescent cells were photographed and counted 1 day after transfection then maintained under conditions of serum deprivation for 14 days (NSPC) or 4 days (SH-SY5Y) and counted relative to cell expressing the control, eGFP tag alone. Histogram represents the mean of the three experiments with SEM. The difference in surviving cell numbers were significant (**,*p* < 0.01 for NSPCs; ****,*p* < 0.0001 for SH-SY5Y cells). **(C)** SH-SY5Y cells were maintained as neurospheres in control proliferation media (Con) or plated on adherent substrate under conditions of serum starvation for 3 days (SS). Protein lysates were processed for western blotting with the indicated antibodies.

During the course of the differentiation experiments, we noticed that expression of the mutant Musashi1 S337A resulted in the survival of more transfected cells than seen with expression of either the wild-type Musashi1 or the control eGFP tag alone. To examine a possible stimulatory effect upon cell survival caused by inhibition of Musashi1 phosphorylation, we exposed the transfected cells to nutrient deprivation in the absence of trophic factors (see Methods). Cell counting indicated that a similar number of Musashi1- or Musashi1 S337A-transfected cells were present, relative to control eGFP-transfected cells, one day after transfection, prior to nutrient deprivation (Figure 4A). However, upon examination of the cells following fourteen days of nutrient deprivation, that resulted in the death of the majority of transfected and un-transfected NSPC cells, we observed that significantly more Musashi1 S337A-transfected cells were present compared to the eGFP control transfected cultures (Figure 4A, Musashi1 S337A; 318.6% number of control cells +/− 6.6% SEM; n = 3). Similarly, after 4 days of nutrient deprivation of SH-SY5Y cells, significantly more Musashi1 S337A-transfected cells survived relative to eGFP-transfected control cells (Figure 4B, Musashi1 S337A; 473% number of control cells +/− 21% SEM; n = 3). Notably, expression of the wild-type Musashi1 protein did not provide a statistically significant survival advantage under the same conditions for either cell type (Figure 4A and B). These results suggest that inhibition of Musashi1 regulatory phosphorylation specifically promotes cell survival under conditions of nutrient deprivation. Consistent with a requirement for Musashi1 regulatory phosphorylation in controlling the cellular response to nutrient deprivation, we observed that Musashi1 S337 phosphorylation increased significantly under conditions of nutrient deprivation (Figure 4C). Over three independent experiments, the normalized ratios of phosphorylated Musashi1 were significantly increased in serum starved over control cells (relative to levels of α-tubulin in the same samples); 1.53, 1.58 and 1.31, respectively with a mean of 1.47 (95% confidence interval: 1.14 to 1.88; p = 0.022, one sample t-test). Consistent with starvation-induced translation of the p21^WAF1/CIP1^ mRNA [33], p21^WAF1/CIP1^ protein levels also increased in SH-SY5Y subject to nutrient deprivation (Figure 4C).

## Discussion

In this study, we show that differentiation and nutrient deprivation modulate Musashi1 function in primary rat NSPC cells. Regulatory phosphorylation of Musashi1 on S337 is correlated with a switch in function from repression to activation of translation of target mRNAs. We show that de-repression of Musashi target mRNAs occurs in the absence of overt loss of Musashi1 protein, indicating that Musashi function is regulated through protein modification rather than degradation, during the initiation of cell fate transitions. Our findings support a model in which Musashi regulation is required for both physiological and pathological neural progenitor cell differentiation and responses to nutrient deprivation. These studies contribute to a growing literature that describes a pivotal role for regulated control of mRNA translation in mediating cell fate transitions [34-36].

A requirement for Musashi regulation during neural cell fate transitions is demonstrated by the inhibitory effect of wild type Musashi1 or phospho-inhibitory Musashi1 S337A mutant protein overexpression in NSPCs and SH-SY5Y cells (Figure 3). Elevated Musashi1 levels are associated with a proliferative predisposition and loss of differentiation potential in a wide variety of tissue and cell types [8,9,11,13,15-17,20]. Conversely, studies utilizing Musashi knockdown strategies have demonstrated that loss of Musashi impedes cell growth and/or survival and promotes differentiation [15,20,37]. Indeed, this is assumed to be the basis for translation of Musashi target mRNAs in mature tissues where Musashi1 levels are very low. Taken together, our findings support a model in which phosphorylation of Musashi, like loss of Musashi protein through knockdown, serves to de-repress and allow translation of target mRNAs. Our contribution here is to reveal a mechanism for control of Musashi function in the absence of reduction in Musashi1 protein levels. Based upon our findings we propose that excess Musashi1 protein inhibits differentiation by functioning as a competitive inhibitor of endogenous Musashi regulation, presumably by saturating the capacity of upstream regulatory mechanisms. Although our observations support a requirement for Musashi1 S337 phosphorylation in mediating differentiation, we cannot exclude the possibility that overexpression of Musashi1 S337 also acts to inhibit differentiation by titrating additional modifying events or essential co-factors [38-40].

A specific role for Musashi1 S337 regulatory phosphorylation was clearly indicated in mediating control of cell growth under conditions of nutrient deprivation as overexpression of the phospho-inhibitory mutant Musashi1 S337A protein specifically promoted survival and growth of both NSPCs and SH-SY5Y cells (Figure 4). By contrast, over-expression of wild-type Musashi1 did not modulate cell survival in nutrient deprived cells (Figure 4). It will be interesting to identify the molecular determinants that distinguish between the effects of wild-type and Musashi1 S337A mutant protein in mediating cell survival. The observation that differentiation, but not responses to nutrient starvation, was inhibited by overexpression of the wild type Musashi1 protein in our system, suggests that a weaker, or more easily competed cellular response drives differentiation compared to nutrient availability.

Regulatory phosphorylation of Musashi1 is necessary for translation of target mRNAs in oocytes and for target mRNA de-repression in a NIH3T3 fibroblast reconstitution assay [31]. We have previously reported that deletion of the C-terminal region of Musashi1 prevents activation of target mRNA translation, with the remaining N-terminal domain acting as a dominant negative inhibitor of target mRNA translation [30]. We concluded that the N-terminal domain, which contains the RNA binding functionality, targets the protein to appropriate mRNA substrates, whereas the C-terminal domain serves to overcome repression and direct translation of the mRNAs, presumably through recruitment of necessary co-associated proteins. We propose that phosphorylation of S337 within the C-terminal domain of Musashi1 may be required to form the active translational induction complex. It is thus anticipated that the non-phosphorylatable mutant Musashi1 acts to enforce repression of target mRNAs. Consistent with enforcement of Musashi1 target mRNA repression promoting survival in our study, Okano and colleagues have recently demonstrated that Musashi1 promotes the survival of human glioma cells through up-regulation of both Notch and PI3 kinase/Akt signaling pathways. This protective effect appears to be mediated in part by repression of Numb and PTEN (an inhibitor of PI3 kinase/Akt signaling) [37]. In addition to p21^WAF1/CIP1^, Numb and PTEN, additional Musashi target mRNAs may contribute to regulation of cell differentiation and cell survival in neural stem and progenitor cell populations [20-22,37,41-43].

To date, the signaling pathways that regulate Musashi1 function in stem and progenitor cells have not been established. We note however, that the conserved regulatory phosphorylation sites in mammalian Musashi1 lie within consensus proline-directed kinase target sequences and both cyclin-dependent kinase and MAP kinase signaling pathways have been directly linked to Musashi1 regulation in oocytes [31]. The highly related Musashi2 protein also contains the sites of regulatory phosphorylation [31]. Since Musashi1 and Musashi2 can act in a redundant manner in neural stem and progenitor cells [44], it is likely that Musashi2 is subject to a similar phosphorylation-dependent regulation during neural differentiation. Together, Musashi1 and Musashi2 have been implicated in the self-renewal of stem and progenitor cells from a number of tissue types [6-8], and we propose that cell fate transitions of multiple stem cell populations may be dependent upon regulated phosphorylation for control of Musashi function.

We observe a low level basal phosphorylation of Musashi1 in proliferating NSPCs and SH-SY5Y cells (Figures 1 and 4). This observation may reflect one of two non-exclusive possibilities: Musashi1 may exist in a dynamic equilibrium between regulated and unregulated function throughout the cell cycle to allow flexibility in the control of target mRNA translation; and/or Musashi1 may be subject to cell cycle phase-specific regulation which appears as a low level basal state in an asynchronous cell population. In either circumstance, the data suggest that even in proliferating cells, a proportion of the Musashi1 protein is regulated and thus promoting translation, rather than repression of target mRNAs. Such a duality in translational control has been reported in proliferating K562 cells where the TGFβR1 mRNA is repressed by Musashi2 while the SMAD3 mRNA is translationally activated by Musashi2 [24]. Taken together, our results indicate that the ability of Musashi to exert repression of target mRNA populations is inversely correlated with site-specific regulatory phosphorylation within the C-terminal domain of the protein. It will be interesting to determine if Musashi mutations that prevent regulatory phosphorylation are associated with loss of cell cycle control in any clinical proliferative disorders.

## Conclusions

Our results reveal that a rapid regulation of Musashi1 function and de-repression and translation of Musashi target mRNAs is required for cell fate transition in response to environmental signals. Our findings suggest that control of physiological and pathological neural stem and progenitor cell self-renewal may be affected by modulating the signaling pathways that control Musashi regulation. Further elucidation of the molecular mechanisms that regulate Musashi function in stem and progenitor cell populations may lead to identification of targets for therapeutic intervention to attenuate pathological Musashi function. Conversely, manipulation of Musashi function may also be employed to maintain Musashi repressor function and promote self-renewal of stem and progenitor cell populations for regenerative medicine.

## Methods

### Plasmid construction

The mouse Musashi-1 protein sequence (GenBank NM_008629) was amplified by PCR from the IMAGE OC29D08 clone. The product was cloned in-frame with the downstream Enhanced Green Flourescent Protein (EGFP) open reading frame using the Xho I and BamH I sites of the pEGFP-N1 plasmid (Clontech) to generate pEGFPN-Msi1. Phosphorylation mutant Musashi protein was generated from pEGFPN-Msi1 as a starting template using site directed mutagenesis (QuikChange, Stratagene, Agilent) to create the S337A mutant. The pcDNAFluc2 reporter plasmid was created through insertion of a Hind III restriction site 128 bp downstream of the BGH poly(A) site in pcDNA3.1(−) (Life Technologies) using the QuikChange kit, followed by plasmid digestion with Hind III and religation to form a modified vector. The Firefly luc2 ORF (Promega) was isolated with Xho I and Eco RI from pCR-Blunt (Life Technologies) and ligated into the Xho I and Eco RI-digested modified pcDNA3.1(−). For luciferase mRNA translation reporter assays we utilized the previously characterized Musashi binding element (MBE) from the *Xenopus* Mos mRNA 3′ UTR [30]. This MBE sequence and a control mRNA sequence from the *Xenopus* β-globin mRNA were PCR amplified out of the pGEM GST β-globin/PRE/MBE and pGEM GST β-globin plasmids, respectively with 5′ BamH I and 3′ Hind III sites. The PCR products were subsequently ligated into BamH I and Hind III-digested pcDNAluc2 to generate pcDNA Fluc-MBE (Fluc-MBE) and pcDNA Fluc β-globin (Fluc-con) respectively [28].

### Cell culture and transfection

Primary neural stem/progenitor cells (NSPCs) were cultured from embryonic rat hippocampal/cortical tissue (Genlantis) and were cultured on low-adhesion plates (Corning) in Neurobasal medium (all cell culture reagents from Life Technologies unless otherwise stated) with B-27 (−vitamin A), 0.5 mM glutamine and 10 μg/ml gentamycin, supplemented with 10 ng/ml bFGF (PeproTech) and 10 ng/ml EGF (PeproTech) to promote proliferation and formation of neurospheres [45]. Prior to use in either proliferation or differentiation experiments neurospheres were dispersed by trypsin treatment and mechanical trituration to produce individual cells and small cell clusters. For differentiation, cells were plated onto poly-D-lysine coated dishes in media lacking bFGF and EGF and supplemented with 1 μM retinoic acid [46]. Extensive neurite extension was apparent within 24 hrs of differentiation. SH-SY5Y human neuroblastoma cells (ATCC) were grown in DMEM, supplemented with 10% fetal calf serum and were induced to differentiate under the same conditions as the NSPCs. Serum starvation-induced cell quiescence was achieved through incubation of cells in DMEM with 0.5% fetal calf serum for three days [47]. Transfections of were performed with Lipofectamine 2000 according to the manufacturer’s instructions. Visualization of EGFP expressing (fluorescent) cells for quantitation of cell number and extent of differentiation was performed using an Olympus IX71 microscope and MetaMorph image analysis software. For cell counting, 5 random fields in each well were photographed and analyzed for cell fluorescence in each replicate experiment (averaging > 100 cells per well). For survival analysis, all fluorescent cells in the well were counted.

### Western blotting

Total cell lysates were prepared in NP-40 lysis buffer supplemented with 1 mM DTT, 0.1 mM PMSF and protease inhibitors [48]. Protein samples (30 μg of total lysates) were denatured and resolved on 10% NuPage gels (Life Technologies) and transferred onto Protran NC membrane (IscBioExpress). Western blots were dried at room temperature for 1 h and blocked with 1% BSA in TBST for 1 h. Primary antibody in 1% BSA and TBST was typically incubated overnight at 4°C (dilution 1:1000), washed, incubated with Anti-rabbit or Anti-mouse IgG HRP conjugate secondary antibody (Promega), and exposed using ChemiGlow substrate on the ChemiImager Imaging System (Alpha Innotech). Antibodies were obtained from Abcam (Musashi-1), Cell Signaling (GAPDH), Millipore (p21) and Sigma (α-tubulin). The generation and characterization of the phospho-specific Musashi1 S337 antibody has been described previously [31]. Relative protein levels were quantitated as previously described [49-51] using AlphaEaseFC software (Alpha Innotech). Representative western blots are shown.

### Luciferase reporter assays

SH-SY5Y cells were co-transfected with the Firefly Luciferase (Fluc) reporter constructs and control Renilla Luciferase plasmid (pRLTK, Promega) and cultured under proliferation or differentiation conditions, as described previously [28]. Luciferase activity was determined after 24 hours, using the Dual-Luciferase Reporter Assay System (Promega) and Turner Biosystems luminometer (Promega) according to the supplier’s protocol. The levels of Firefly luciferase reporter mRNA were determined using semi-quantitative PCR. No significant differences in stability of the Fluc-Con or Fluc-MBE mRNAs were detected under the conditions employed.

### Statistical analyses

Quantitated data are presented either as mean +/− SEM, or as mean and 95% confidence interval (CI). The 95% CI is simply calculated as the mean +/− t*SEM, where *t* is the 97.5 percentile from the appropriate t-distribution. Student’s two tailed, paired *t*-tests were used to analyze the data, where *p* < 0.05 was adopted for statistical significance. Where indicated, statistical significance was also assessed by one way ANOVA with Bonferroni multiple comparison test and in each case a probability of *p* < 0.05 was adopted for statistical significance.

## Abbreviations

NSPC: Neural stem/progenitor cell
MBE: Musashi binding element
SEM: Standard error of the mean
eGFP: Enhanced green fluorescent protein
PI3 Kinase: Phosphatidylinositol-3-kinase
PTEN: Phosphatase and tensin homolog
Akt: Protein Kinase B
GAPDH: Glyceraldehyde 3-phosphate dehydrogenase

## References

[1] Young RA (2011). Control of the embryonic stem cell state. Cell.

[2] Orkin SH, Wang J, Kim J, Chu J, Rao S, Theunissen TW (2008). The transcriptional network controlling pluripotency in ES cells. Cold Spring Harb Symp Quant Biol.

[3] Torres-Padilla ME, Chambers I (2014). Transcription factor heterogeneity in pluripotent stem cells: a stochastic advantage. Development.

[4] Cahan P, Daley GQ (2013). Origins and implications of pluripotent stem cell variability and heterogeneity. Nat Rev Mol Cell Biol.

[5] Sarkar A, Hochedlinger K (2013). The sox family of transcription factors: versatile regulators of stem and progenitor cell fate. Cell Stem Cell.

[6] de Andres-Aguayo L, Varas F, Graf T (2012). Musashi 2 in hematopoiesis. Curr Opin Hematol.

[7] MacNicol AM, Wilczynska A, MacNicol MC (2008). Function and regulation of the mammalian Musashi mRNA translational regulator. Biochem Soc Trans.

[8] Okano H, Kawahara H, Toriya M, Nakao K, Shibata S, Imai T (2005). Function of RNA-binding protein Musashi-1 in stem cells. Exp Cell Res.

[9] Hemmati HD, Nakano I, Lazareff JA, Masterman-Smith M, Geschwind DH, Bronner-Fraser M (2003). Cancerous stem cells can arise from pediatric brain tumors. Proc Natl Acad Sci U S A.

[10] Ito T, Kwon HY, Zimdahl B, Congdon KL, Blum J, Lento WE (2010). Regulation of myeloid leukaemia by the cell-fate determinant Musashi. Nature.

[11] Kanemura Y, Mori K, Sakakibara S, Fujikawa H, Hayashi H, Nakano A (2001). Musashi1, an evolutionarily conserved neural RNA-binding protein, is a versatile marker of human glioma cells in determining their cellular origin, malignancy, and proliferative activity. Differentiation.

[12] Kharas MG, Lengner CJ, Al-Shahrour F, Bullinger L, Ball B, Zaidi S (2010). Musashi-2 regulates normal hematopoiesis and promotes aggressive myeloid leukemia. Nat Med.

[13] Oskarsson T, Acharyya S, Zhang XH, Vanharanta S, Tavazoie SF, Morris PG (2011). Breast cancer cells produce tenascin C as a metastatic niche component to colonize the lungs. Nat Med.

[14] Schiapparelli P, Enguita-German M, Balbuena J, Rey JA, Lazcoz P, Castresana JS (2010). Analysis of stemness gene expression and CD133 abnormal methylation in neuroblastoma cell lines. Oncol Rep.

[15] Sureban SM, May R, George RJ, Dieckgraefe BK, McLeod HL, Ramalingam S (2008). Knockdown of RNA binding protein musashi-1 leads to tumor regression in vivo. Gastroenterology.

[16] Toda M, Iizuka Y, Yu W, Imai T, Ikeda E, Yoshida K (2001). Expression of the neural RNA-binding protein Musashi1 in human gliomas. Glia.

[17] Wang XY, Penalva LO, Yuan H, Linnoila RI, Lu J, Okano H (2010). Musashi1 regulates breast tumor cell proliferation and is a prognostic indicator of poor survival. Mol Cancer.

[18] Wang XY, Yu H, Linnoila RI, Li L, Li D, Mo B (2013). Musashi1 as a potential therapeutic target and diagnostic marker for lung cancer. Oncotarget.

[19] Yokota N, Mainprize TG, Taylor MD, Kohata T, Loreto M, Ueda S (2004). Identification of differentially expressed and developmentally regulated genes in medulloblastoma using suppression subtraction hybridization. Oncogene.

[20] Battelli C, Nikopoulos GN, Mitchell JG, Verdi JM (2006). The RNA-binding protein Musashi-1 regulates neural development through the translational repression of p21(WAF-1). Mol Cell Neurosci.

[21] Horisawa K, Imai T, Okano H, Yanagawa H (2009). 3′-Untranslated region of doublecortin mRNA is a binding target of the Musashi1 RNA-binding protein. FEBS Lett.

[22] Imai T, Tokunaga A, Yoshida T, Hashimoto M, Mikoshiba K, Weinmaster G (2001). The neural RNA-binding protein Musashi1 translationally regulates mammalian numb gene expression by interacting with its mRNA. Mol Cell Biol.

[23] Spears E, Neufeld KL (2011). Novel double-negative feedback loop between adenomatous polyposis coli and Musashi1 in colon epithelia. J Biol Chem.

[24] Park SM, Deering RP, Lu Y, Tivnan P, Lianoglou S, Al-Shahrour F (2014). Musashi-2 controls cell fate, lineage bias, and TGF-beta signaling in HSCs. J Exp Med.

[25] Erhardt JA, Pittman RN (1998). Ectopic p21WAF1 expression induces differentiation-specific cell cycle changes in PC12 cells characteristic of nerve growth factor treatment. J Biol Chem.

[26] Hughes AL, Gollapudi L, Sladek TL, Neet KE (2000). Mediation of nerve growth factor-driven cell cycle arrest in PC12 cells by p53. Simultaneous differentiation and proliferation subsequent to p53 functional inactivation. J Biol Chem.

[27] Yan G, Ziff EB (1995). NGF regulates the PC12 cell cycle machinery through specific inhibition of the Cdk kinases and induction of cyclin D1. J Neuroscience.

[28] MacNicol MC, Cragle CE, MacNicol AM (2011). Context-dependent regulation of Musashi-mediated mRNA translation and cell cycle regulation. Cell Cycle.

[29] Arumugam K, Wang Y, Hardy LL, MacNicol MC, MacNicol AM (2010). Enforcing temporal control of maternal mRNA translation during oocyte cell cycle progression. EMBO J.

[30] Charlesworth A, Wilczynska A, Thampi P, Cox LL, MacNicol AM (2006). Musashi regulates the temporal order of mRNA translation during Xenopus oocyte maturation. EMBO J.

[31] Arumugam K, MacNicol MC, Wang Y, Cragle CE, Tackett AJ, Hardy LL (2012). Ringo/CDK and MAP kinase regulate the activity of the cell fate determinant Musashi to promote cell cycle re-entry in Xenopus oocytes. J Biol Chem.

[32] Warfel NA, El-Deiry WS (2013). p21WAF1 and tumourigenesis: 20 years after. Curr Opin Oncol.

[33] Shi Y, Felley-Bosco E, Marti TM, Orlowski K, Pruschy M, Stahel RA (2012). Starvation-induced activation of ATM/Chk2/p53 signaling sensitizes cancer cells to cisplatin. BMC Cancer.

[34] Heinrich EM, Dimmeler S (2012). MicroRNAs and stem cells: control of pluripotency, reprogramming, and lineage commitment. Circ Res.

[35] Biedermann B, Hotz HR, Ciosk R (2010). The Quaking family of RNA-binding proteins: coordinators of the cell cycle and differentiation. Cell Cycle.

[36] Bitterman PB, Polunovsky VA (2012). Translational control of cell fate: from integration of environmental signals to breaching anticancer defense. Cell Cycle.

[37] Muto J, Imai T, Ogawa D, Nishimoto Y, Okada Y, Mabuchi Y (2012). RNA-binding protein Musashi1 modulates glioma cell growth through the post-transcriptional regulation of Notch and PI3 kinase/Akt signaling pathways. PLoS One.

[38] Kawahara H, Okada Y, Imai T, Iwanami A, Mischel PS, Okano H (2011). Musashi1 cooperates in abnormal cell lineage protein 28 (Lin28)-mediated let-7 family microRNA biogenesis in early neural differentiation. J Biol Chem.

[39] Cragle C, Macnicol AM (2014). Musashi-directed translational activation of target mRNAs is mediated by the poly[A] polymerase, Germline Development-2. J Biol Chem.

[40] Bish R, Vogel C (2014). RNA binding protein-mediated post-transcriptional gene regulation in medulloblastoma. Mol Cells.

[41] de Sousa Abreu R, Sanchez-Diaz PC, Vogel C, Burns SC, Ko D, Burton TL (2009). Genomic analyses of musashi1 downstream targets show a strong association with cancer-related processes. J Biol Chem.

[42] Vo DT, Subramaniam D, Remke M, Burton TL, Uren PJ, Gelfond JA (2012). The RNA-binding protein Musashi1 affects medulloblastoma growth via a network of cancer-related genes and is an indicator of poor prognosis. Am J Pathol.

[43] Rutledge CE, Lau HT, Mangan H, Hardy LL, Sunnotel O, Guo F (2014). Efficient translation of Dnmt1 requires cytoplasmic polyadenylation and Musashi binding elements. PLoS One.

[44] Sakakibara S, Nakamura Y, Yoshida T, Shibata S, Koike M, Takano H (2002). RNA-binding protein Musashi family: roles for CNS stem cells and a subpopulation of ependymal cells revealed by targeted disruption and antisense ablation. Proc Natl Acad Sci U S A.

[45] Nakamura Y, Sakakibara S, Miyata T, Ogawa M, Shimazaki T, Weiss S (2000). The bHLH gene hes1 as a repressor of the neuronal commitment of CNS stem cells. J Neurosci.

[46] Cao QL, Howard RM, Dennison JB, Whittemore SR (2002). Differentiation of engrafted neuronal-restricted precursor cells is inhibited in the traumatically injured spinal cord. Exp Neurol.

[47] Davis PK, Ho A, Dowdy SF (2001). Biological methods for cell-cycle synchronization of mammalian cells. Biotechniques.

[48] MacNicol AM, Muslin AJ, Williams LT (1993). Raf-1 kinase is essential for early Xenopus development and mediates the induction of mesoderm by FGF. Cell.

[49] Charlesworth A, Cox LL, MacNicol AM (2004). Cytoplasmic polyadenylation element (CPE)- and CPE-binding protein (CPEB)-independent mechanisms regulate early class maternal mRNA translational activation in xenopus oocytes. J Biol Chem.

[50] Charlesworth A, Ridge JA, King LA, MacNicol MC, MacNicol AM (2002). A novel regulatory element determines the timing of Mos mRNA translation during Xenopus oocyte maturation. EMBO J.

[51] Charlesworth A, Welk J, MacNicol A (2000). The temporal control of Wee1 mRNA translation during *Xenopus* oocyte maturation is regulated by cytoplasmic polyadenylation elements within the 3′ untranslated region. Dev Biol.

